# Using the photoplethysmography method to monitor age-related changes in the cardiovascular system

**DOI:** 10.3389/fphys.2023.1191272

**Published:** 2023-07-19

**Authors:** Biljana Djurić, Katarina Žikić, Zorica Nestorović, Danijela Lepojević-Stefanović, Nebojša Milošević, Dejan Žikić

**Affiliations:** ^1^ Institute of Physiology, Faculty of Medicine, University of Belgrade, Belgrade, Serbia; ^2^ Faculty of Physics, University of Belgrade, Belgrade, Serbia; ^3^ Institute of Biophysics, Faculty of Medicine, University of Belgrade, Belgrade, Serbia; ^4^ Division of Cardiology, Department of Internal Medicine, Belgrade, Serbia

**Keywords:** photoplethysmography, sensor, cardiovascular age, blood flow waveform, detrended fluctuation analysis

## Abstract

**Introduction:** Aging is a physiological process characterized by progressive changes in all organ systems. In the last few decades, the elderly population has been growing, so the scientific community is focusing on the investigation of the aging process, all in order to improve the quality of life in elderly. One of the biggest challenges in studying the impact of the aging on the human body represents the monitoring of the changes that inevitably occur in arterial blood vessels. Therefore, the medical community has invested a great deal of effort in studying and discovering new methods and tools that could be used to monitor the changes in arterial blood vessels caused by the aging process. The goal of our research was to develop a new diagnostic method using a photoplethysmographic sensor and to examine the impact of the aging process on the cardiovascular system in adults. Long-term recorded arterial blood flow waveforms were analyzed using detrended fluctuation analysis.

**Materials and Methods:** The study included 117 respondents, aged 20–70 years. The waveform of the arterial blood flow was recorded for 5 min, with an optical sensor placed above the left common carotid artery, simultaneously with a single-channel ECG. For each cardiac cycle, the blood flow amplitude was determined, and a new time series was formed, which was analyzed non-linearly (DFA method). The values of the scalar coefficients *α*_1_ and *α*_2_, particularly their ratio (*α*_1_/*α*_2_) were obtained, which were then monitored in relation to the age of the subjects.

**Result:** The values of the scalar ratio (*α*_1_/*α*_2_) were significantly different between the subjects older and younger than 50 years. The value of the *α*_1_/*α*_2_ decreased exponentially with the aging. In the population of middle-aged adults, this ratio had a value around 1, in young adults the value was exclusively higher than 1 and in older adults the value was exclusively lower than 1.

**Conclusion:** The results of this study indicated that the aging led to a decrease in the *α*_1_/*α*_2_ in the population of healthy subjects. With this non-invasive method, changes in the cardiovascular system due to aging can be detected and monitored.

## 1 Introduction

Human life has evidently prolonged over the last 50 years, but cardiovascular diseases are still the leading cause of mortality in the modern world (Rudnicka et al., 2020; World Health Organization, 2021). The research that addresses the causes of the disease, age-related changes of blood vessels and the analysis of blood flow waveforms are highly important for patients benefit (Gavrilov and Gavrilova, 2015; Van Leeuwen et al., 2019; Rudnicka et al., 2020).

The cardiovascular system changes through years, particularly it changes its biophysical properties: increase in arterial blood pressure and pulse wave velocity, as well as the appearance of wave reflection (Parikh et al., 2016; Guzik and Touyz, 2017; Allen et al., 2020). Moreover, according to some cardiologists, the older your cardiovascular system is, the older you are (Dantas et al., 2012; Gopcevic et al., 2021; Hamczyk et al., 2022).

The proper medical diagnosis as well as the cardiovascular research, depends from biophysical understanding of arterial hemodynamics, especially from pressure and blood flow waveforms conditions (Willemet and Alastruey, 2015; Jozwiak et al., 2018). More precisely, the blood viscosity, elasticity of the arterial wall, the thickness of the wall, the internal pressure, the blood density, the influence of gravity and body position can be recognized as main biophysical characteristics of blood propagation in vessel (Secomb, 2016; Žikić et al., 2019).

Pulse wave velocity (PWV) measurement is still the primary method for assessing the age of arteries in clinical research (Sutton-Tyrrell et al., 2005; Reference Values for Arterial Stiffness' Collaboration, 2010; Diaz et al., 2018). Previous studies show that the PWV depends on the elasticity of the artery wall, wall thickness, wall diameter and blood density (Avolio, 2013; Messas et al., 2013; Ma et al., 2018).

The main goal of the present study is to find a new biophysical model that could monitor the aging of the cardiovascular system. This idea was created by following the arterial blood flow, with non-invasive measurement of the arterial blood flow waveform. There are two basic techniques of non-invasive blood flow measurement: ultrasonographic and optical. The first method is very sensitive (depending on the experience and skills of medical professionals) and cannot provide reliable signals for wave analysis (Srámek et al., 2000; Loizou, 2014). On the other hand, the second method is independent from the muscle’s activity, although there is a problem with the signal calibration (Žikić, 2008; Djuric et al., 2017).

Although the main goal of this study is the analysis of the distribution of wave blood flow with age, the comparison of the mean values of scalar coefficients in three selected age groups can be considered as a secondary goal. Nevertheless, although implicitly presented, the essential goal of this study is to present a non-invasive method of imaging arterial blood flow by photoplethysmography method. In addition, a mathematical model that follows this kind of signal analysis (*Detrended fluctuation analysis - DFA*) can reliably estimate the arterial blood flow waveform in the cardiovascular system during aging.

## 2 Materials and methods

### 2.1 Subject, groups, and physical examination

The study was conducted at the Faculty of Medicine, University of Belgrade (Serbia), from September 2019 to April 2021 at the Institute of Medical physiology and the Institute of Biophysics. The study was carried out following the recommendations of the Helsinki Committee, which is confirmed by the decision of the Ethics Committee of the Faculty of Medicine.

In total, 117 healthy subjects (58 male and 59 female), aged between 20 and 70 years, have participated in this study. Based on the age in which the frequency of vascular disease symptoms increases markedly, the subjects were divided into two groups (Lloyd-Jones et al., 2006; Wang et al., 2020; Mohanty et al., 2021): younger than 50 years (82) and older than 50 years (35). In addition, subjects have been divided into three age groups (Petry, 2002; Thomas et al., 2018) according to the demographic characteristics. The first group was the younger adults (53) less than 35 years, then middle-aged adults (44) from 35 to 55 years. Finally, the third group was older adults (22) higher than 55 years.

### 2.2 Signal recording

For the purposes of this research, the photoplethysmography sensor designed and developed earlier (Žikić, 2008), has been used for continuous and non-invasive recording of the arterial blood flow waveform (Djuric et al., 2017). The sensor was designed in order to eliminate artifacts that were occurring during the phases of respiration, caused by the contraction of the respiratory muscles. In the improved design, the light source was two series-connected IR diodes, and the detector was three series-connected NPN phototransistors. Three serially connected silicon NPN phototransistors were used as light intensity change detectors (Djuric et al., 2017).

**FIGURE 1:**
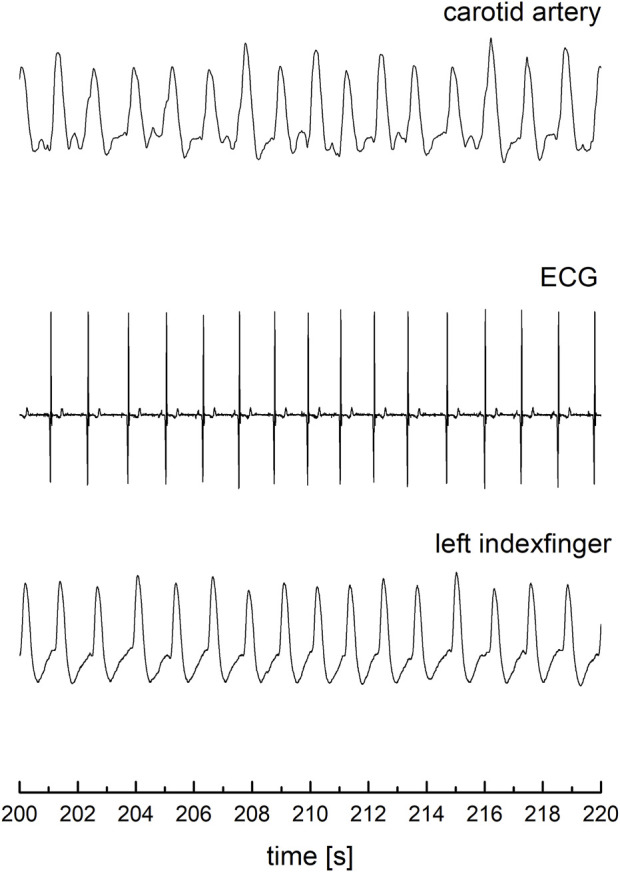
Recorded ECG and blood flow waveforms from the left index finger and the carotid artery.

The photoplethysmography sensor was placed and secured over the left common carotid artery (Vlachopoulos et al., 2010). Synchronized with the recording of carotid arterial blood flow waveform, a single-lead channel ECG was also recorded, using three electrodes on the surface of the chest, as well as arterial blood flow waveform on the left index finger in order to detect artifacts due to movement of the neck, swallowing or breathing. (Figure 1). Recordings were performed in the supine position for 5–7 min, in order to provide a long-lasting signal suitable for further mathematical analysis (Figure 1).

**FIGURE 2:**
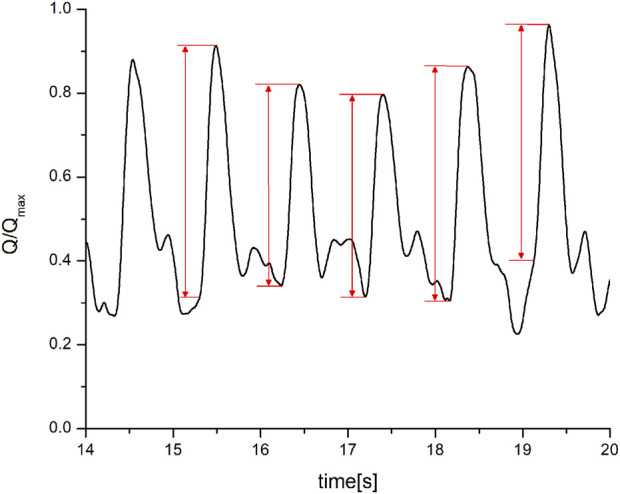
Determination of the maximal and minimal value of the arterial blood flow waveform (Q/Q_max_–flow amplitude normalized in relation to the maximum amplitude.

All the signals from the sensor module and ECG device were digitized in 12-bit resolution (PCI-20428W, data-acquisition board, United States) with a sampling frequency of 1 Khz. After smoothing and normalization of the signal (by dividing the whole signal by the maximum measured value - Q/Qmax), the amplitudes of the arterial blood flow waveform were determined for each cardiac cycle, for a minimum of 256 heartbeats. Amplitudes were calculated as the difference between the maximal reached value of the waveform and the foot-of-the-wave (Figure 2).

**FIGURE 3:**
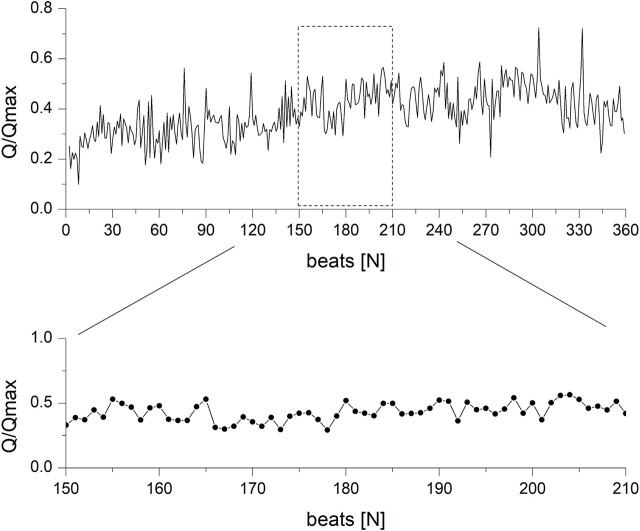
Amplitude of the arterial blood flow waveform in the function of the number of heartbeats.

Based on the obtained difference values, a new signal, amplitude in function of the heartbeat number N, was constructed (Figure 3).

### 2.3 Data processing

For each subject, the recording of the arterial flow was analyzed by determining the amplitudes (*z*_*i*_) for each heartbeat (Peng et al., 1995). Then, on the entire signal, the extracted amplitudes were made as a function of the number of beats (*n*_*i*_), i.e., *z*_*i*_ = *f* (*n*_*i*_). The sequence was further analyzed using the nonlinear dynamics technique, applied to physiological signals, i.e., detrended fluctuation analysis or DFA (Peng et al., 1995; Hausdorff et al., 1996). According to this technique the sequence *y*(*k*) was formed (Hardstone et al., 2012) and divided into segments of non-overlapping length *n*). In each segment the linear local trend (*y*_*n*_(*k*)) was calculated (Peng et al., 1995; Hardstone et al., 2012).

In the next step, the root mean square of the fluctuations in series *y*_*n*_ on all *N* segments of length *n* was calculated (Peng et al., 1995; Bryce and Sprague, 2012). In case when the relationship *F*(*n*) vs *n* is some sort of power function (for instance, const⋅*n* ^*α*^) the graph is shown by a straight line whose direction coefficient is *α*.

**FIGURE 4:**
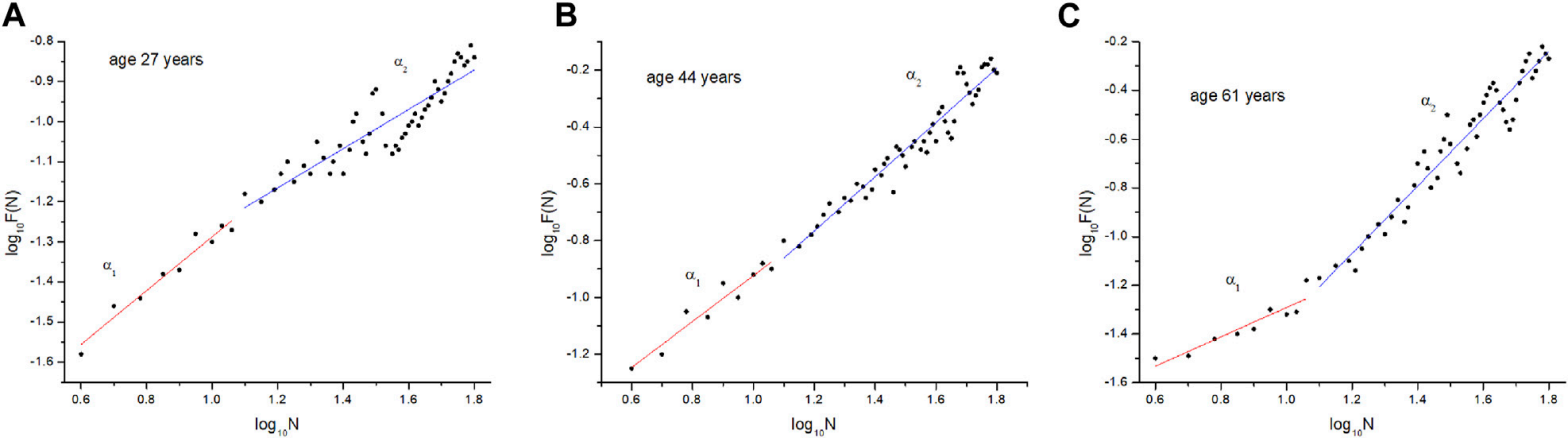
Results of arterial blood-flow waveform fluctuations in young **(A)**, middle-aged **(B)**, and elderly **(C)** subject.

The DFA method applied in this way defines two coefficients: *α*_1_ and *α*_2_. The first refers to correlations over short distances, while the second defines correlations over long temporal distances. In this study, *α*_1_ is the slope of the linear fit between 4 and 15 beats, while *α*_2_ is the slope of the linear fit more than 15 heart beats (Figure 4). At the end, the ratio of the slope *α*_1_ */α*_2_, as the result of the entire signal analysis, was established.

### 2.4. Statistics

Monitoring and signal registration was performed using the LabView software (National Instruments Corp. United States). Further processing of the recorded signal and graphical presentation was done in OriginPro 8.0 (OriginLab). At the end, the statistical evaluation of the measured values was carried out using SPSS 26 (IBM, United States, demo version).

Taking into account the number of values within each age group (Table 1), the statistical evaluation between the mean values was performed using the *t*-test for independent samples (A-B), as well as the Mann-Whitney test between two pairs (A-C and B-C).

**TABLE 1:** The number of subjects and the corresponding *α*_1_/*α*_2_ statistical parameters (mean, standard deviation *SD*, standard error *SE* and range) in three age groups (A, B and C). Symbol * indicates level of statistical difference between groups.

Group	A	B	C
Number (*n*)	53	44	22
Mean	1.24	0.87 ***(A)	0.67***(A,B)
SD	0.19	0.18	0.09
SE	0.03	0.03	0.02
Range	0.69	0.94	0.33

## 3 Results

The DFA analysis was performed after several subjects were measured for over 20 min in order to obtain 1,024 points. However, the preliminary results suggested that the time interval could be shortened for two reasons: 1) after 7–8 min of recording the subjects became uncomfortable and started moving their head/neck involuntarily, and 2) the same outcome (in the DFA analysis) were obtained with 256 points. Thus, the measurement session lasted 5 min.

**FIGURE 5:**
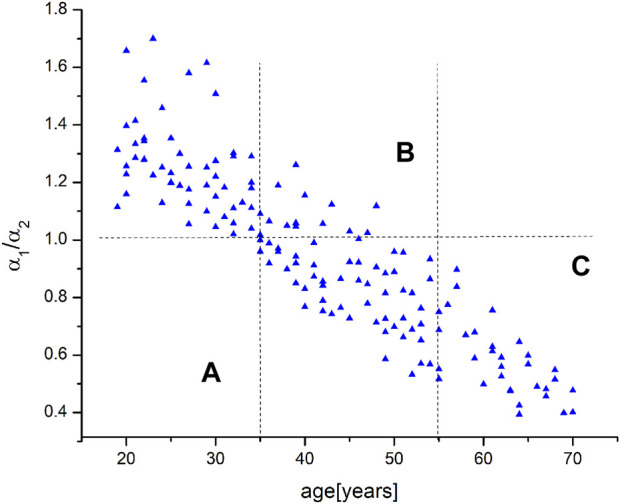
The relationship between α_1_/α_2_ and the age of all subjects who participated in the study. The vertical lines define intervals in the three age groups **(A–C)**.

Figure 5 shows the distribution of the *α*_1_/*α*_2_ with age, from 20 to 70 years. The vertical lines show the distribution of the *α*_1_/*α*_2_ values across the three age groups of subjects (A, B and C). Qualitative analysis of Figure 5 shows that in the first group, the values of the *α*_1_/*α*_2_ are greater than 1, i.e., they range from 1 to 1.7. At the same time, it can be seen that the *α*_1_/*α*_2_ values for group 2 oscillate around the number 1 (from 0.5 to 1.3). It is possible to conclude that the majority of the *α*_1_/*α*_2_ are below 1, but this was only the case of this study. Finally, the third group of subjects reliably has values of the *α*_1_/*α*_2_ less than 1 (between 0.4 and 1.0).

The next step involved finding the function that most accurately fits the data. The resulting distribution was fitted with four functions: linear (-*a*⋅(age) + *b*), logarithmic (*a*⋅ln (age) + *b*), exponential (*a*⋅exp (-*b*⋅age)), and polynomial (*a*⋅(age)^2^ - *b*⋅(age) + *c*). The quality of fitting was examined by comparing the correlation coefficients *R*) and comparing the residuals in relation to the fitted curve. The results showed that the polynomial residuals adjust the curve to the data more than they fit it. On the other hand, the exponential function has the highest *R* (0.841) in comparison with the logarithmic and linear functions (0.813 and 0.809, respectively). Thus, the nature of the distribution should be presented with the exponential function.

Table 1 shows the calculated *α*_1_/*α*_2_ values of the statistical parameters (mean value, standard deviation, standard error, and range) for the three age groups of the subjects. The symbol *n* indicates the number of subjects in each group, making it clear that comparison of means requires the use of nonparametric statistics. Considering the number of samples, the statistical analysis of the mean values of the first and second groups was performed using the *t*-test for two independent samples. On the other hand, the comparison of mean values between other pairs (A-C and B-C) was carried out using the Mann-Whitney test. The results showed that the mean values of all pairs showed a convincing statistical significance (*p* < 0.001, Table 1).

## 4 Discussion

The morphological and functional changes that are occurring in the cardiovascular system during aging are known as the Vascular Health Triad (Townsend et al., 2015). These processes lead to the appearance of arteriosclerosis and atherosclerosis (Diaz et al., 2018). To be precise, an increase in the stiffness of the wall leads to an increase in arterial blood pressure and pulse wave velocity (Ranadive et al., 2021). DFA of signal fluctuation represents the analysis of self-similarity of the fractal structure in relation to the signal as a whole (Peng et al., 1995). This kind of analysis is applicable to various physiological parameters. By using DFA of signal fluctuations, there is a possibility of finding the long-term correlations within the chaotic values in physiological signals (Esen et al., 2009).

Previous studies have shown that the DFA is primarily used in heart-rate analysis (Peng et al., 1995; De Souza et al., 2014; Mizobuchi et al., 2021). However, the existence of amplitude fluctuations for certain parameter, in relation to the time domain, has been only demonstrated in the analysis of continuous EEG recordings (Hardstone et al., 2012; De Souza et al., 2014; Facioli et al., 2021; Mizobuchi et al., 2021). The amplitude of the recorded signal shows large fluctuations (Eke et al., 2002), but the amplitude values can be characterized by the scalar coefficient *α*). The value of an *α* value higher than 0.5 shows a positive correlation in fluctuation of the recorded signal. Conversely, the value smaller than 0.5 indicates the absence of correlation, i.e., the existence of small fluctuations (Hardstone et al., 2012).

The present study examines the possibilities of using the DFA technique in the analysis of fluctuations in the amplitude of blood pulse waves. To the best of our knowledge, no study has been reported that analyzes the amplitude fluctuations of arterial blood flow waveforms of subjects of different ages. Therefore, this study represents a novelty in the application of DFA, but considering the number of subjects, the study is still only preliminary. In addition, the study included subjects of different ages (between 20 and 70 years of age) without heart rhythm abnormalities and other cardiac or vascular diseases. The subjects were classified into three age groups (Section 2.1) mostly for the reason of indirectly examining the difference in the morphology of the artery walls (Djuric, 2022). Namely, the difference in the wall morphology with age was quantified through the difference in the fluctuations of the amplitude of the blood flow waveform (Djuric, 2022).

The quantification of the fluctuations of the amplitudes of arterial blood flow waveform was performed by determining the scalar coefficients (*α*_1_ and *α*_2_) over the years, or more precisely their ratio (*α*_1_/*α*_2_). The results of this study clearly show that the ratio of the scalar coefficients decreases with age (Figure 4). This distribution was best represented by the exponential function. Further analysis of the distribution showed that the selected age groups differed significantly from each other (Table 1). In this study, a significant difference between subjects over 55 years (Group C), both in relation other groups (Group A and B) could be seen as expected result. However, the highly significant difference between Group B and C could be seen as a novelty. Subjects with higher values of *α*_1_/*α*_2_ demonstrated the existence of a correlation in waveform amplitude through the entire photoplethysmography signal (Eke et al., 2000; Eke al., 2002; Hardstone et al., 2012). Large fluctuations in the amplitude are most likely occurring due to the high elasticity of the arterial vessels (Zarrinkoob et al., 2016).

Further, the decrease of the *α*_1_/*α*_2_ with age may indicate a decrease in the fluctuation of the waveform amplitude, and this probably occurs because of changes in the elasticity of the wall of the arterial blood vessel (Mitchel, 2021). Finally, observing the distribution of the *α*_1_/*α*_2_ in relation to the age, and comparing the values between different age groups, we can conclude that the mentioned ratio represents a sensitive marker for assessing the age of the vascular system in the population of healthy subjects.

Assessing the age of the cardiovascular system is very difficult to determine with non-invasive measurement methods. With aging, changes occur in the walls of blood vessels, which will certainly affect the waveform of the blood flow. Validation of this method is very difficult. We plan to repeat the measurement of the same subjects after a shorter period and compare the results of the analysis. Another limitation of the method is the impact of hypertension and blood viscosity on the blood flow waveform. These parameters will be included in further studies.

## 5 Conclusion

Cardiovascular diseases are the leading cause of mortality in Serbia and the rest of the world (Institute of Public Health of Serbia, 2020; National Center for Health Statistics, 2021; Tsao et al., 2023). The research which addresses age-related changes of blood vessels, particularly the analysis of blood flow waveforms, is highly important for patients’ benefit. This becomes especially important knowing that in the last 20 years the number of old people has been growing.

Reviewing the present literature (Charlton et al., 2021), we did not notice a study that analyzes the long-term arterial blood flow waveform within different age ranges. It is usually studied by ultrasonographic analysis of blood flow, so this study represents a novelty by finding that blood flow waveforms recorded by photoplethysmography based sensor can be used for the research of the cardiovascular system aging. However, considering the number of subjects and their age range, we perceive this study as preliminary. As such, this study registers an exponential decrease in the ratio of the scalar coefficients with age and detects differences between the mean values of their ratio in the three selected age groups. In addition, our analysis could indicate certain changes in the cardiovascular system that cannot be detected by standard non-invasive methods.

Therefore, we believe that our new analysis of wave propagation of blood may be very useful for indication of cardiovascular diseases. Our analysis method could contribute to faster diagnosis and adequate therapeutic decision for a better quality of life for older people (American Heart Association, 2019; Lindbohm et al., 2019; Neumann et al., 2022).

## Data Availability

The original contributions presented in the study are included in the article/Supplementary Material, further inquiries can be directed to the corresponding author.
